# Supplementary material for: Characterization of a triad of genes in cyanophage S-2L sufficient to replace adenine by 2-aminoadenine in bacterial DNA
Source: Nat Commun. 2021 Aug 5;12:4710. doi: 10.1038/s41467-021-25064-x (PMC8342488; doi:10.1038/s41467-021-25064-x)
Supplement: Supplementary file 1 — Supplementary Information [file 41467_2021_25064_MOESM1_ESM.pdf]

Characterization of a triad of genes in cyanophage S-2L sufficient to replace adenine by 2-aminoadenine in bacterial DNA

Dariusz Czernecki, Frédéric Bonhomme,
Pierre-Alexandre Kaminski and Marc Delarue

Supplementary Information

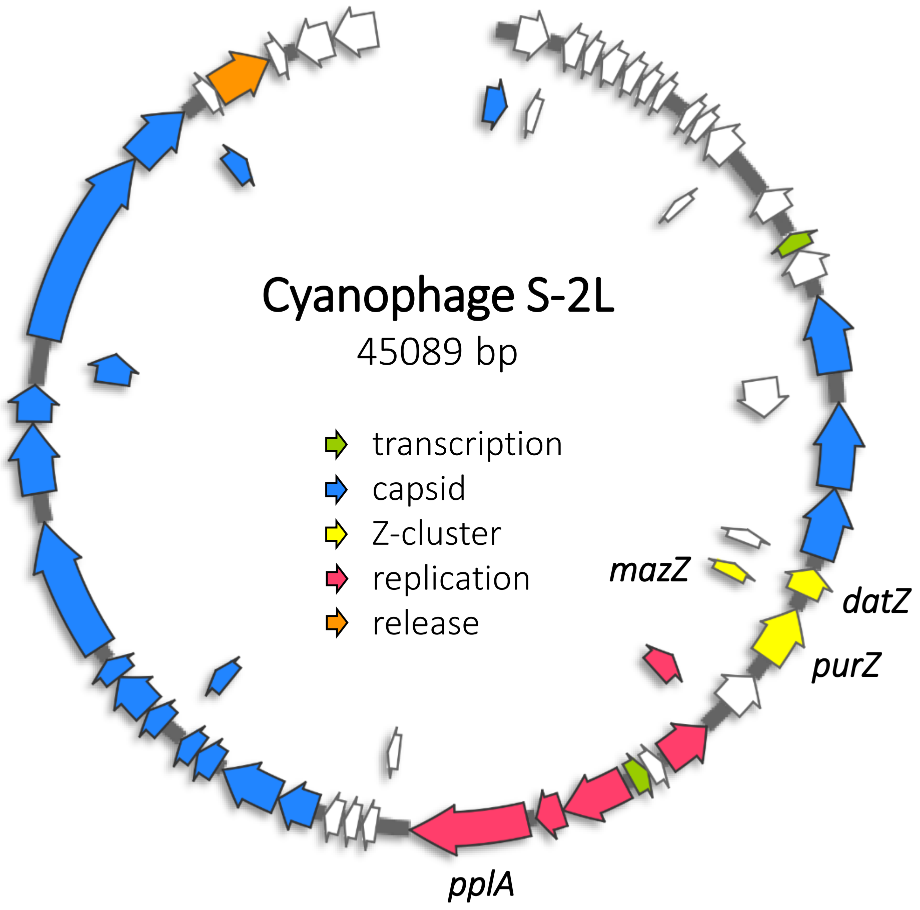

Supplementary Figure 1. Map of the S-2L genome. All identified genes (arrows) are coloured according to their function, as indicated inside the circle. Specifically indicated are the Z-cluster genes (*purZ*, *mazZ*, *datZ*), along with the gene of PrimPol (*pplA*).

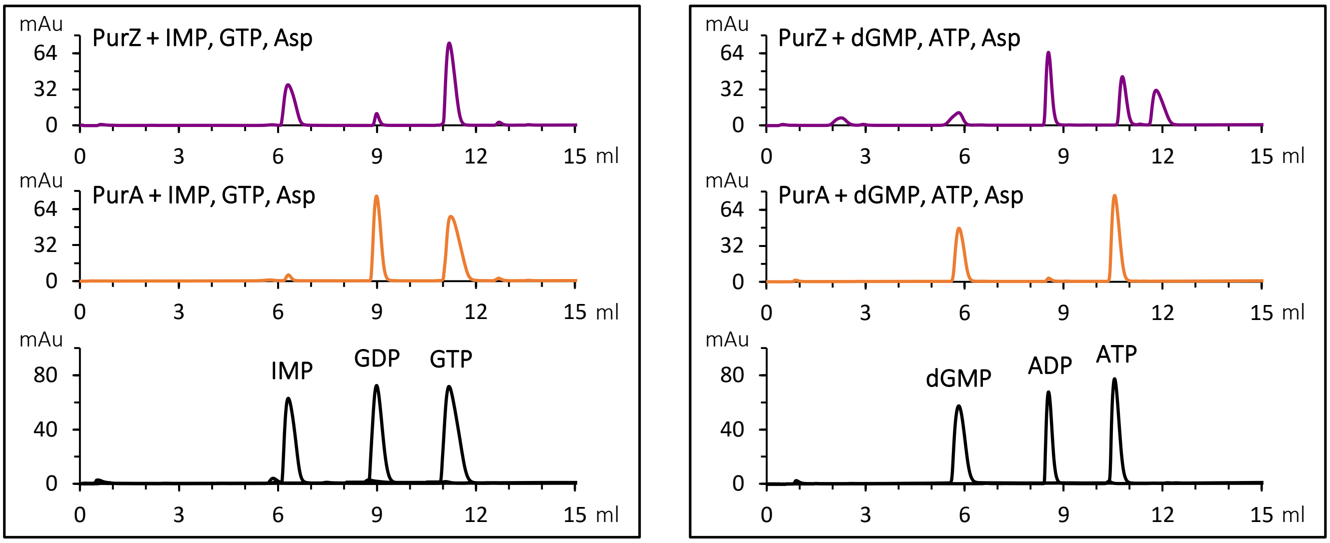

Supplementary Figure 2. Comparison of the enzymatic activities of S-2L PurZ and *E. coli* PurA, visualised with HPLC plots. Nucleotide standards are shown below in black. In the reaction time of 15 min, PurA rapidly transforms IMP, GTP and Asp mixture into GDP and sIMP, whereas PurZ stays inactive even at higher concentration (left panel). Inversely, PurZ catalyses the reaction from dGMP, ATP and Asp to ADP and dSMP, contrary to PurA that does not recognise these substrates (right panel). Although the sIMP peak is confounded with the GTP one, it gives a noticeable shift in 260/280 nm absorbance ratio.

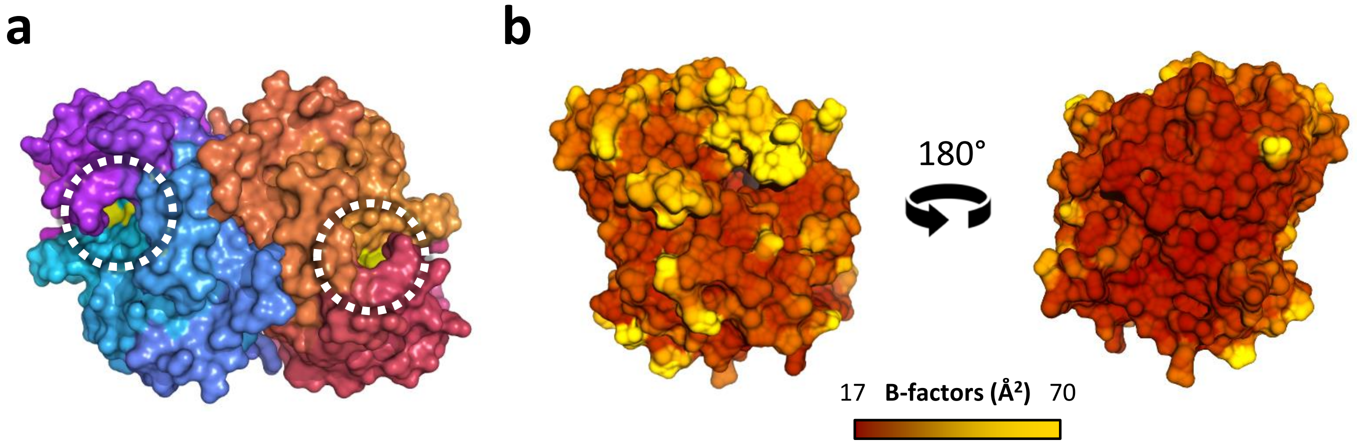

Supplementary Figure 3. Properties of the S-2L PurZ structure highlighting its dimeric state. a PurZ dimer, in surface representation: the two subunits are coloured in cyan-purple and orange-light red gradients. White dotted circles point to the opposite catalytic sites. **b** Surface representation of PurZ coloured using experimental B-factors, with the corresponding scale bar below. The flexible loop above the catalytic cleft (left) define the aspartate loop. The interface between the dimer's subunits (right) is particularly rigid, strongly suggesting a constitutive dimeric form of PurZ.

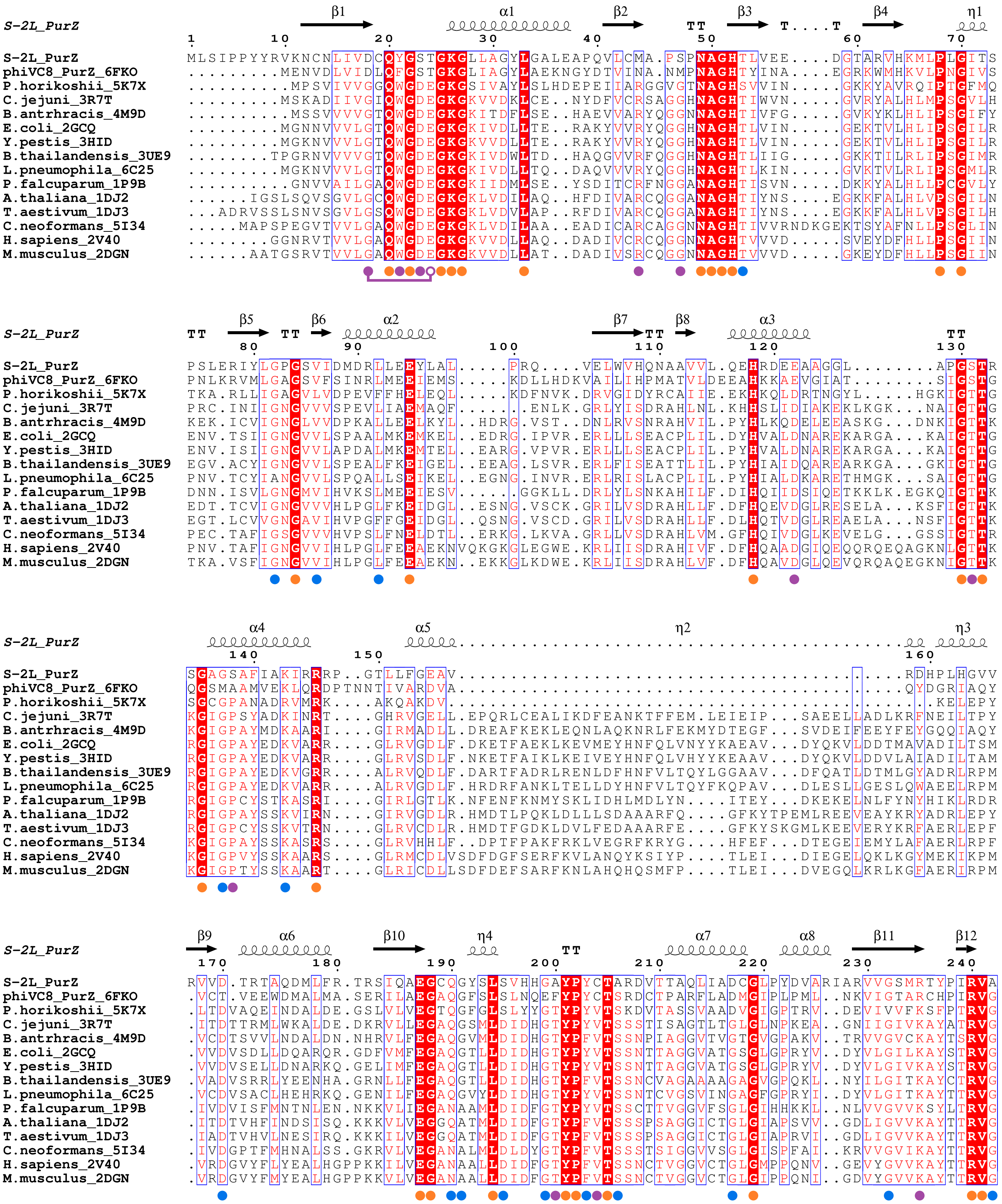

S-2L_PurZ
β1 α1 β2 TT β3 T....T β4 η1
1 10 20 30 40 50 60 70
S-2L_PurZ MLSIPPYYRVKNCNLIVDCQYGSTGKGLLAGYLGALEAPQVLCMA.PSPNAGHTLVEE....DGTARVHKMLPLGITS
phiVC8_PurZ_6FKO .........MENVDLVIDLQFGSTGKGLIAGYLAEKNGYDTVINA.NMPNAGHTYINA....EGRKWMHKVLPNGIVS
P.horikoshii_5K7X ..........MPSVIVVGGQWGDEGKGSIVAYLSLHDEPEIIARGGVGTNAGHSVVIN.....GKKYAVRQIPTGFMQ
C.jejuni_3R7T .........MSKADIIVGIQWGDEGKGKVVDKLCE..NYDFVCRSAGGHNAGHTIWVN....GVRYALHLMPSGVLH
B.antrhracis_4M9D ..........MSSVVVVGTQWGDEGKGKITDFLSE..HAEVVARYQGGNNAGHTIVFG....GVKYKLHLIPSGIFY
E.coli_2GCQ .........MGNNVVVLGTQWGDEGKGKIVDLLTE..RAKYVVRYQGGHNAGHTLVIN....GEKTVLHLIPSGILR
Y.pestis_3HID .........MGKNVVVLGTQWGDEGKGKVVDLLTE..RAKYVVRYQGGHNAGHTLVIN....GEKTVLHLIPSGILR
B.thailandensis_3UE9 .......TPGRNVVVVGTQWGDEGKGKIVDWLTD..HAQGVVRFQGGHNAGHTLIIG....GKKTILRLIPSGIMR
L.pneumophila_6C25 .........MGKNVVVLGTQWGDEGKGKIVDLLTQ..DAQVVVRYQGGHNAGHTLKIN....GVKTVLRLIPSGMLR
P.falcuparum_1P9B ..........GNVVAILGAQWGDEGKGKIIDMLSE..YSDITCRFNGGANAGHTISVN....DKKYALHLLPCGVLY
A.thaliana_1DJ2 ......IGSLSQVSGVLGCQWGDEGKGKLVDILAQ..HFDIVARCQGGANAGHTIYNS....EGKKFALHLVPSGILN
T.aestivum_1DJ3 ...ADRVSSLSNVSGVLGSQWGDEGKGKLVDVLAP..RFDIVARCQGGANAGHTIYNS....EGKKFALHLVPSGILH
C.neoformans_5I34 .....MAPSPEGVTVVLGAQWGDEGKGKLVDILAA..EADICARCAGGNNAGHTIVVRNDKGEKTSYAFNLLPSGLIN
H.sapiens_2V40 .......GGNRVTVVLGAQWGDEGKGKVVDLLAQ..DADIVCRCQGGNNAGHTVVVD....SVEYDFHLLPSGIIN
M.musculus_2DGN ......AATGSRVTVVLGAQWGDEGKGKVVDLLAT..DADIVSRCQGGNNAGHTVVVD....GKEYDFHLLPSGIIN
S-2L_PurZ
TT β5 TT β6 α2 β7 TT β8 α3 TT
80 90 100 110 120 130
S-2L_PurZ PSLERIYLGPGSVIDMDRLLEEYLAL.....PRQ....VELWVHQNAAVVL.QEHRDEEAAGGL........APGSTR
phiVC8_PurZ_6FKO PNLKRVMLGAGSVFSINRLMEEIEMS.....KDLLHDKVAILIHPMATVLDEEAHKKAEVGIAT.......SIGSTG
P.horikoshii_5K7X TKA.RLLIGAGVLVDPEVFFHELEQL.....KDFNVK.DRVGIDYRCAIIE.EKHKQLDRTNGYL.....HGKIGTTG
C.jejuni_3R7T PRC.INIIGNGVVVSPEVLIAEMAQF.....ENLK.GRLYISDRAHLNL.KHHSLIDIAKEKLKGK...NAIGTTG
B.antrhracis_4M9D KEK.ICVIGNGLVVDPKALLEELKYL..HDRG.VST..DNLRVSNRAHVIL.PYHLKQDELEEASKG...DNKIGTTK
E.coli_2GCQ ENV.TSIIGNGVVLSPAALMKEMKEL..EDRG.IPVR.ERLLLSEACPLIL.DYHVALDNAREKARGA...KAIGTTG
Y.pestis_3HID ENV.ISIIGNGVVLAPDALMKEMTEL..EARG.VPVR.ERLLLSEACPLIL.PYHVALDNAREKARGA...KAIGTTG
B.thailandensis_3UE9 EGV.ACYIGNGVVLSPEALFKEIGEL..EEAG.LSVR.ERLFISEATTLIL.PYHIAIDQAREARKGA...GKIGTTG
L.pneumophila_6C25 PNV.TCYIANGVVLSPQALLSEIKEL..EGNG.INVR.ERLRISLACPLIL.PYHIALDKARETHMGK...SAIGTTG
P.falcuparum_1P9B DNN.ISVLGNGMVIHVKSLMEEIESV.....GGKLL.DRLYLSNKAHILF.DIHQIIDSIQETKKLK.EGKQIGTTK
A.thaliana_1DJ2 EDT.TCVIGNGVVVHLPGLFKEIDGL..ESNG.VSCK.GRILVSDRAHLLF.DFHQEVDGLRESELA...KSFIGTTK
T.aestivum_1DJ3 EGT.LCVVGNGAVIHVPGFFGEIDGL..QSNG.VSCD.GRILVSDRAHLLF.DLHQTVDGLREAELA...NSFIGTTK
C.neoformans_5I34 PEC.TAFIGSGVVVHVPSLFNELDTL..ERKG.LKVA.GRLLVSDRAHLVM.GFHQIVDGLKEVELG...GSSIGTTR
H.sapiens_2V40 PNV.TAFIGNGVVIHLPGLFEEAEKNVQKGKGLEGWE.KRLIISDRAHIVF.DFHQAADGIQEQQRQEQAGKNLGTTK
M.musculus_2DGN TKA.VSFIGNGVVIHLPGLFEEAEKN..EKKGLKDWE.KRLIISDRAHLVF.DFHQAVDGLQEVQRQAQEGKNIGTTK
S-2L_PurZ
α4 α5 η2 η3
140 150 160
S-2L_PurZ SGAGSAFIAKIRRRP..GTLLFGEAV...................................RDHPLHGVV
phiVC8_PurZ_6FKO QGSMAAMVEKLQRDPTNNTIVARDVA...................................QYDGRIAQY
P.horikoshii_5K7X SGCGPANADRVMRK....AKQAKDV.....................................KELEPY
C.jejuni_3R7T KGIGPSYADKINRT....GHRVGELL.EPQRLCEALIKDFEANKTFFEM.LEIEIP...SAEELLADLKRFNEILTPY
B.antrhracis_4M9D KGIGPAYMDKAARI....GIRMADLL.DREAFKEKLEQNLAQKNRLFEKMYDTEGF...SVDEIFEEYFEYGQQIAQY
E.coli_2GCQ RGIGPAYEDKVARR....GLRVGDLF.DKETFAEKLKEVMEYHNFQLVNYYKAEAV...DYQKVLDDTMAVADILTSM
Y.pestis_3HID RGIGPAYEDKVARR....GLRVSDLF.NKETFAIKLKEIVEYHNFQLVHYYKEAAV...DYQKVLDDVLAIADILTAM
B.thailandensis_3UE9 RGIGPAYEDKVGRR....ALRVQDLF.DARTFADRLRENLDFHNFVLTQYLGGAAV...DFQATLDTMLGYADRLRPM
L.pneumophila_6C25 RGIGPAYEDKVARR....ALRVGDLF.HRDRFANKLTELLDYHNFVLTQYFKQPAV...DLESLLGESLQWAEELRPM
P.falcuparum_1P9B RGIGPCYSTKASRI....GIRLGTLK.NFENFKNMYSKLIDHLMDLYN....ITEY...DKEKELNLFYNYHIKLRDR
A.thaliana_1DJ2 RGIGPCYSSKVIRN....GIRVGDLR.HMDTLPQKLDLLLSDAAARFQ....GFKYTPEMLREEVEAYKRYADRLEPY
T.aestivum_1DJ3 RGIGPCYSSKVTRN....GLRVCDLR.HMDTFGDKLDVLFEDAAARFE....GFKYSKGMLKEEVERYKRFAERLEPF
C.neoformans_5I34 KGIGPAYSSKASRS....GLRVHHLF..DPTFPAKFRKLVEGRFKRYG....HFEF...DTEGEIEMYLAFAERLRPF
H.sapiens_2V40 KGIGPVYSSKAARS....GLRMCDLVSDFDGFSERFKVLANQYKSIYP....TLEI...DIEGELQKLKGYMEKIKPM
M.musculus_2DGN KGIGPTYSSKAART....GLRICDLLSDFDEFSARFKNLAHQHQSMFP....TLEI...DVEGQLKRLKGFAERIRPM
S-2L_PurZ
β9 α6 β10 η4 TT α7 α8 β11 β12
170 180 190 200 210 220 230 240
S-2L_PurZ RVVD.TRTAQDMLFR.TRSIQAEGCQGYSLSVHHGAYPYCTARDVTTAQLIADCGLPYDVARIARVVGSMRTYPIRVA
phiVC8_PurZ_6FKO .VCT.VEEWDMALMA.SERILAEGAQGFSLSLNQEFYPYCTSRDCTPARFLADMGIPLPML..NKVIGTARCHPIRVG
P.horikoshii_5K7X .LTDVAQEINDALDE.GSLVLVEGTQGFGLSLYYGTYPYVTSKDVTASSVAADVGIGPTRV..DEVIVVFKSFPTRVG
C.jejuni_3R7T .ITDTTRMLWKALDE.DKRVLLEGAQGSMLDIDHGTYPFVTSSSTISAGTLTGLGLNPKEA..GNIIGIVKAYATRVG
B.antrhracis_4M9D .VCDTSVVLNDALDN.NHRVLFEGAQGVMLDIDHGTYPFVTSSNPIAGGVTVGTGVGPAKV..TRVVGVCKAYTSRVG
E.coli_2GCQ .VVDVSDLLDQARQR.GDFVMFEGAQGTLLDIDHGTYPYVTSSNTTAGGVATGSGLGPRYV..DYVLGILKAYSTRVG
Y.pestis_3HID .VVDVSELLDNARKQ.GELIMFEGAQGTLLDIDHGTYPYVTSSNTTAGGVATGSGLGPRYV..DYVLGIVKAYSTRVG
B.thailandensis_3UE9 .VADVSRRLYEENHA.GRNLLFEGAQGTLLDIDHGTYPYVTSSNCVAGAAAAGAGVGPQKL..NYILGITKAYCTRVG
L.pneumophila_6C25 .VCDVSACLHEHRKQ.GENILFEGAQGVYLDIDHGTYPYVTSSNTCVGSVINGAGFGPRYI..DYVLGITKAYCTRVG
P.falcuparum_1P9B .IVDVISFMNTNLEN.NKKVLIEGANAAMLDIDFGTYPYVTSSCTTVGGVFSGLGIHHKKL..NLVVGVVKSYLTRVG
A.thaliana_1DJ2 .ITDTVHFINDSISQ.KKKVLVEGGQATMLDIDFGTYPFVTSSSPSAGGICTGLGIAPSVV..GDLIGVVKAYTTRVG
T.aestivum_1DJ3 .IADTVHVLNESIRQ.KKKILVEGGQATMLDIDFGTYPFVTSSSPSAGGICTGLGIAPRVI..GDLIGVVKAYTTRVG
C.neoformans_5I34 .IVDGPTFMHNALSS.GKRVLVEGANALMLDLDYGTYPFVTSSSTSIGGVVSGLGISPFAI..KRVVGVIKAYTTRVG
H.sapiens_2V40 .VRDGVYFLYEALHGPPKKILVEGANAALLDIDFGTYPFVTSSNCTVGGVCTGLGMPPQNV..GEVYGVVKAYTTRVG
M.musculus_2DGN .VRDGVYFMYEALHGPPKKVLVEGANAALLDIDFGTYPFVTSSNCTVGGVCTGLGIPPQNI..GDVYGVVKAYTTRVG

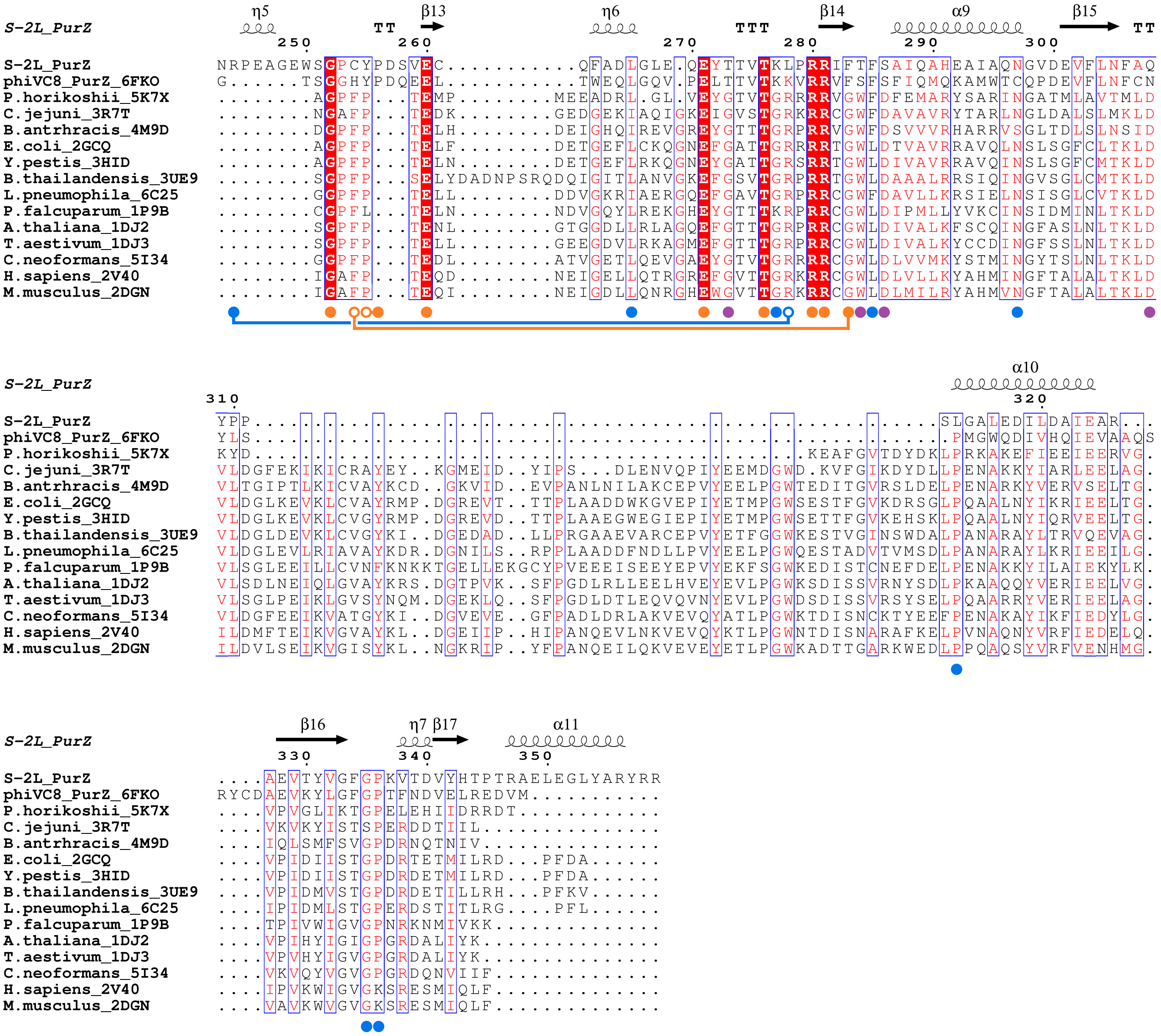

Supplementary figure 4. Structural multialignment between S-2L PurZ and all 14 homologous synthetases available in the PDB. Organism names and PDB codes are indicated on the left. Full circles below the alignment mark positions of residues of interest, divided into three categories: residues strictly conserved among all PurA/PurZ representatives (orange); loosely conserved residues, with two possible variants in both PurA and PurZ (blue); residues strictly conserved in PurA, but not in PurZ enzymes (purple). Empty circles highlight conserved residues with sequence rearrangements: these have shifted backbone position with respect to S-2L's ones (connected full circles), but superposing functional groups. Occasional unstructured and unbuilt regions were filled with sequence information.

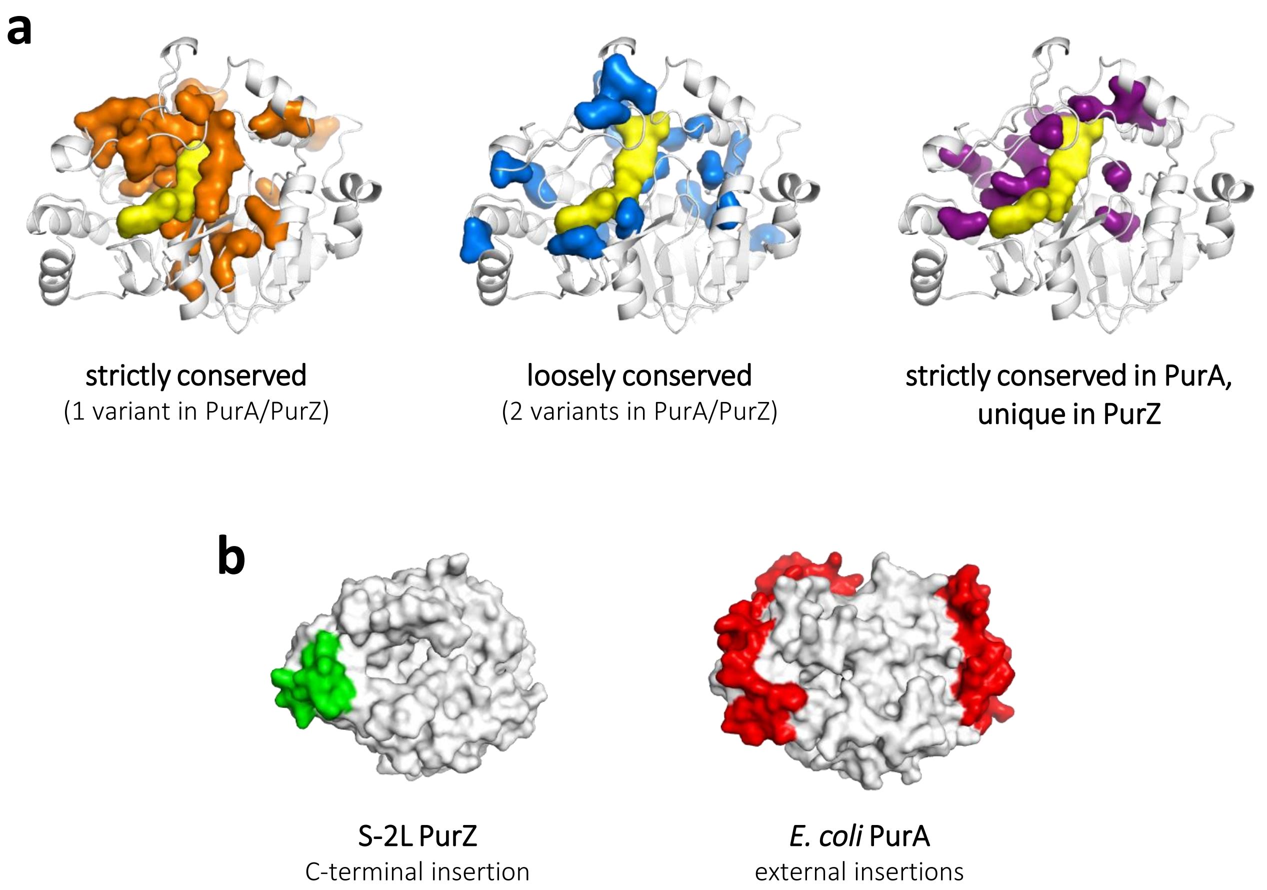

Supplementary Figure 5. Visualisation of the relationships between S-2L PurZ and its structural homologues. a Conserved residues from Supplementary Figure 4 are mapped onto the S-2L PurZ structure, using the same colour code. Nucleotide substrates – dGMP and dATP – are in yellow. **b** Visualisation of indels using surface representation. The S-2L-specific C-terminal insertion in form of an alpha helix (green) is shown on the left. On the right, two large insertions present in bacteria and eukaryotes (red) are mapped on *E.coli* PurA; they are absent in archaeal PurA and viral PurZ.

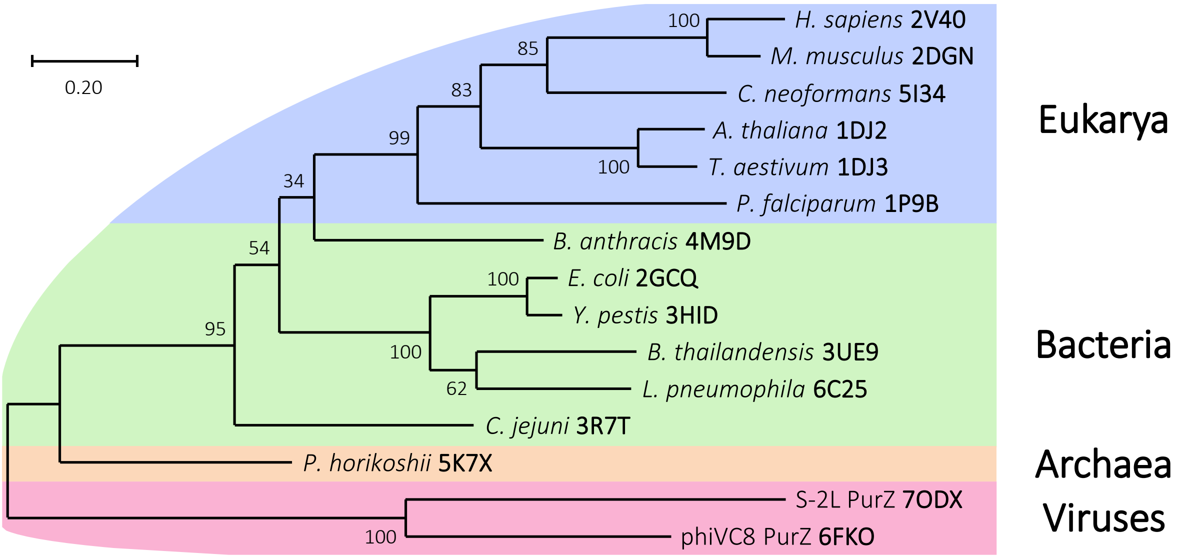

Supplementary Figure 6. Non-rooted maximum-likelihood phylogenetic tree of PurA/PurZ synthetases. The tree was generated using the structural alignment from Supplementary Figure 4. The enzymes are divided into four clades: eukaryotic, bacterial, archaeal and viral. Archaeal PurA is structurally the closest relative of viral PurZ. The reference distance corresponds to an average 0.2 substitution per site. The topology of the bootstrap consensus tree is identical, supporting the result presented here.

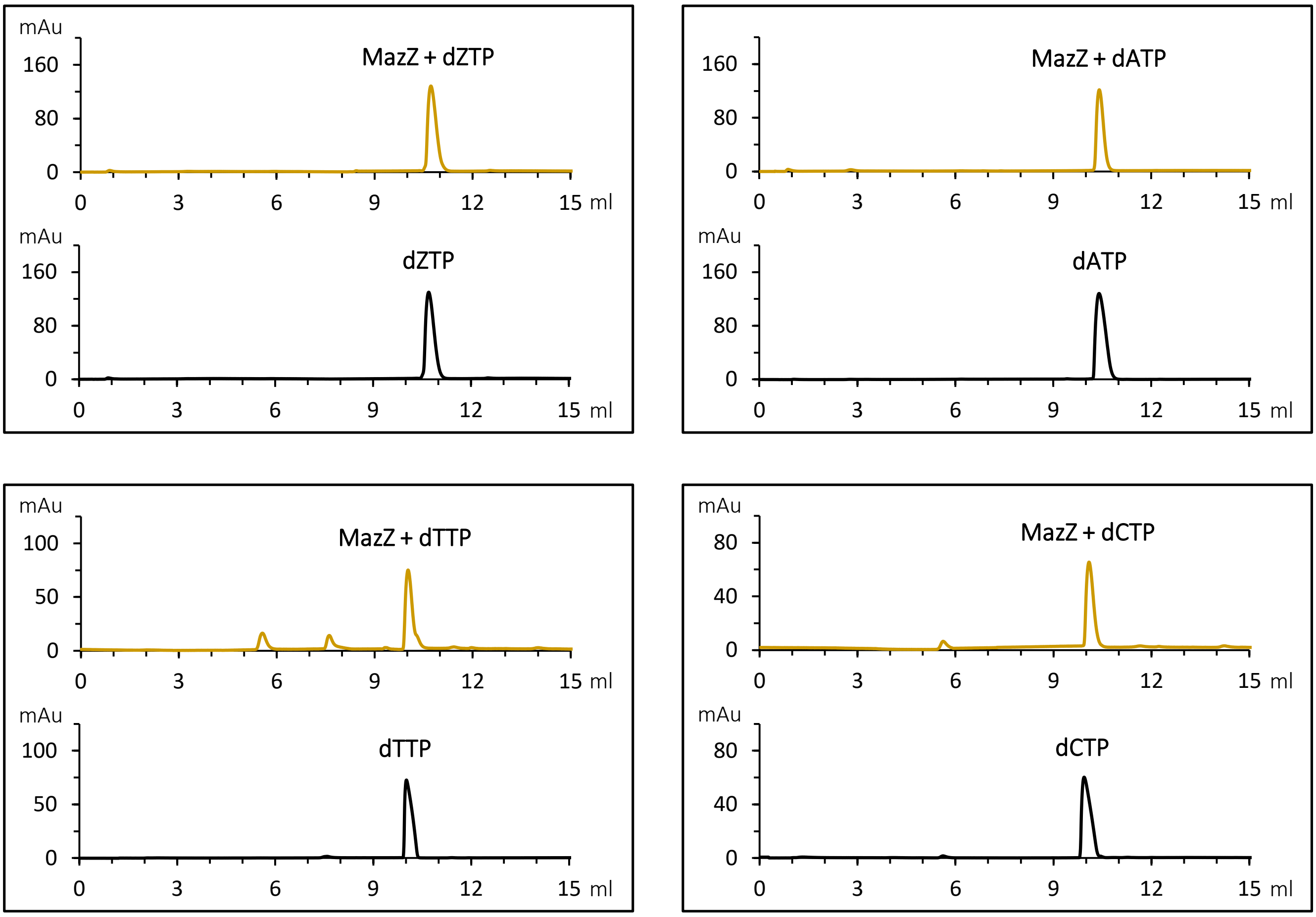

Supplementary Figure 7. HPLC profiles showing lack of S-2L MazZ specificity for deoxynucleoside triphosphates other than dGTP, including dZTP. Nucleotide standards are shown in black; similar profiles are obtained after incubation of the corresponding triphosphates with MazZ (in gold).

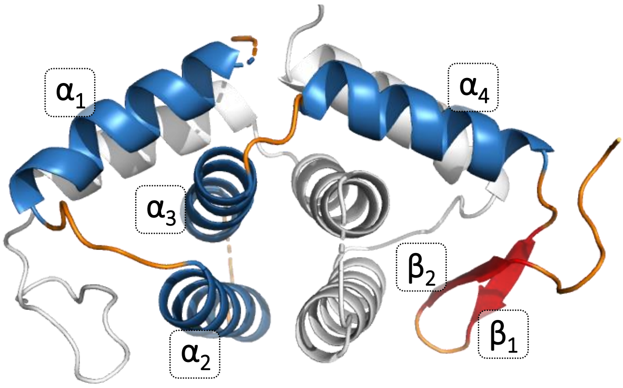

S-2L MazZ
7ODY

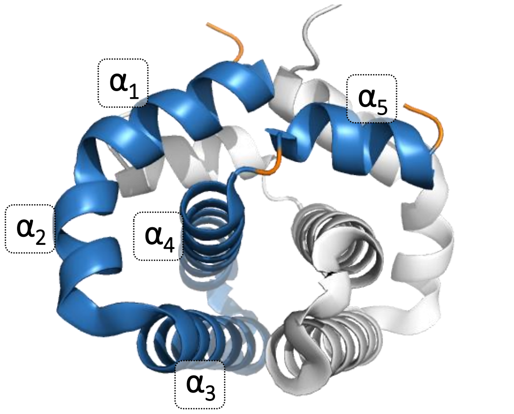

S. solfataricus MazG
1VMG

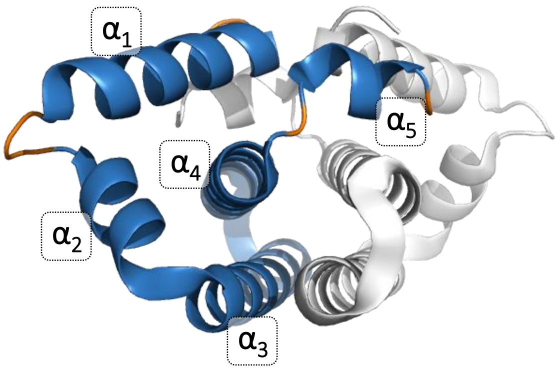

M. tuberculosis HisE
1Y6X

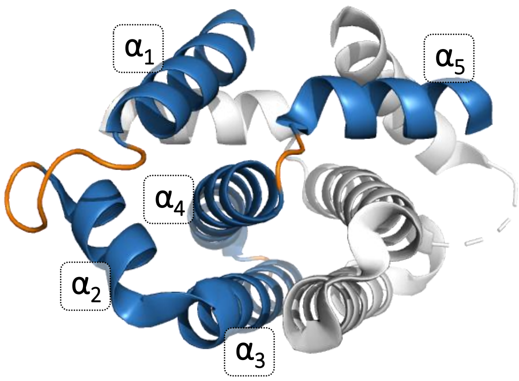

S. flexneri HisIE (HisE domain)
6J2L

Supplementary Figure 8. Common fold shared by S-2L MazZ and bacterial MazG and HisE proteins. The tight dimer part is shown for four exemplary structures, viewed from the same perspective. For each enzyme both chains are shown in ribbon representation. One of the chains is coloured: α-helices in blue, loops in orange and β-strands in red. For the coloured chain, the secondary structure elements are numbered. Below each image, the organism of origin, protein name and the PDB code are indicated.

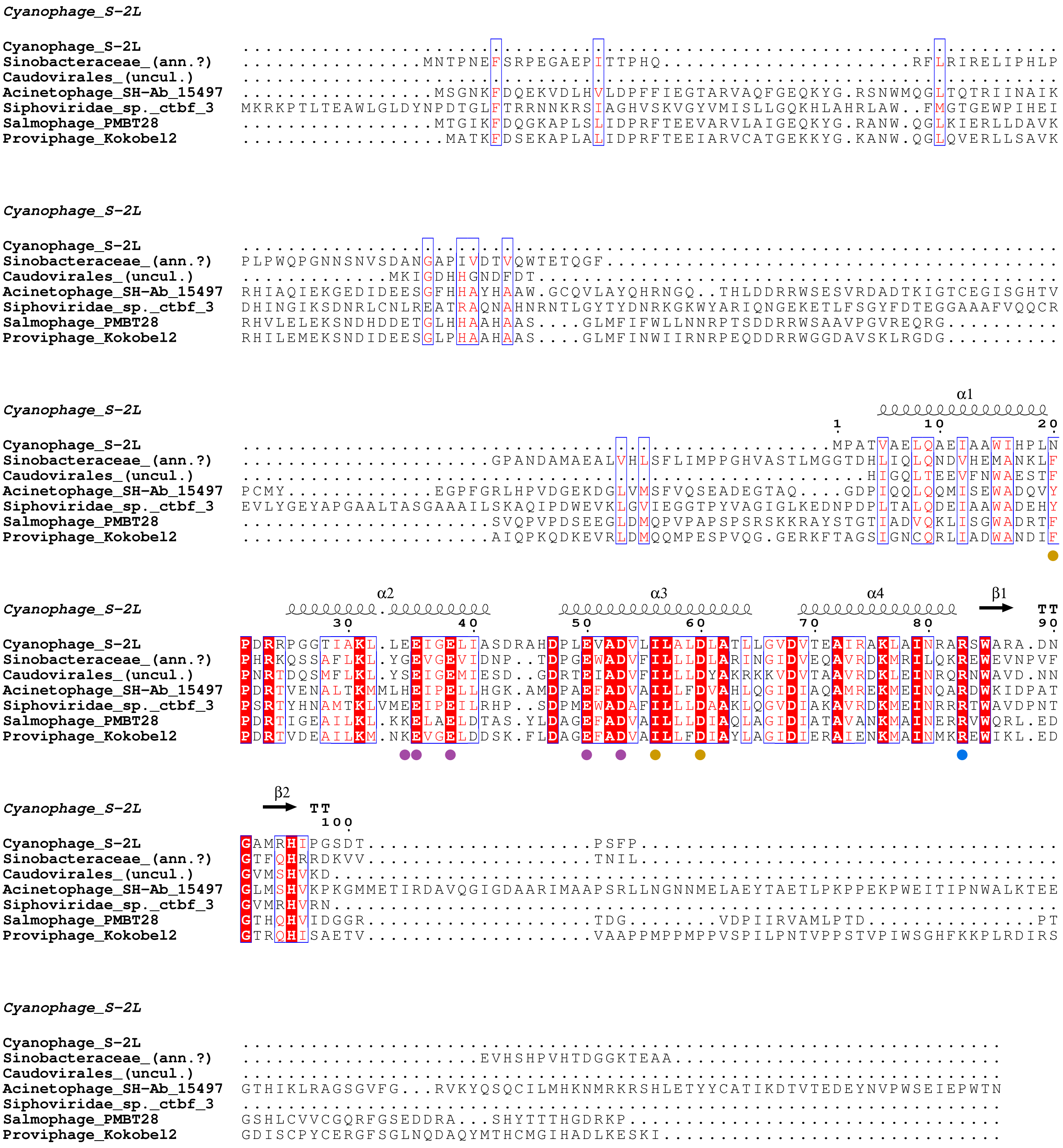

Supplementary Figure 9. Multiple sequence alignment of MazZ-1 homologues (S-2L-like). Proteins from recently sequenced phages were additionally included. Numbering above the alignment refers to S-2L MazZ. Marked below are the catalytic residues of S-2L MazZ (purple dots), residues coordinating guanine nucleobase (orange) and R83 stabilising the reaction intermediate (blue).

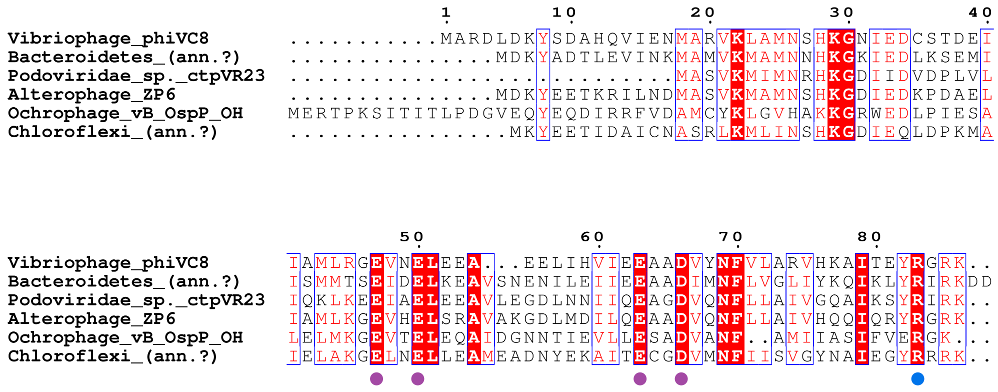

Supplementary Figure 10. Multiple sequence alignment of MazZ-2 homologues (ϕVC8-like). Numbering above the alignment refers to ϕVC8 MazZ. Putative catalytic residues are marked with purple and blue dots, corresponding to S-2L MazZ E35, E38, E50, D53 and R83.

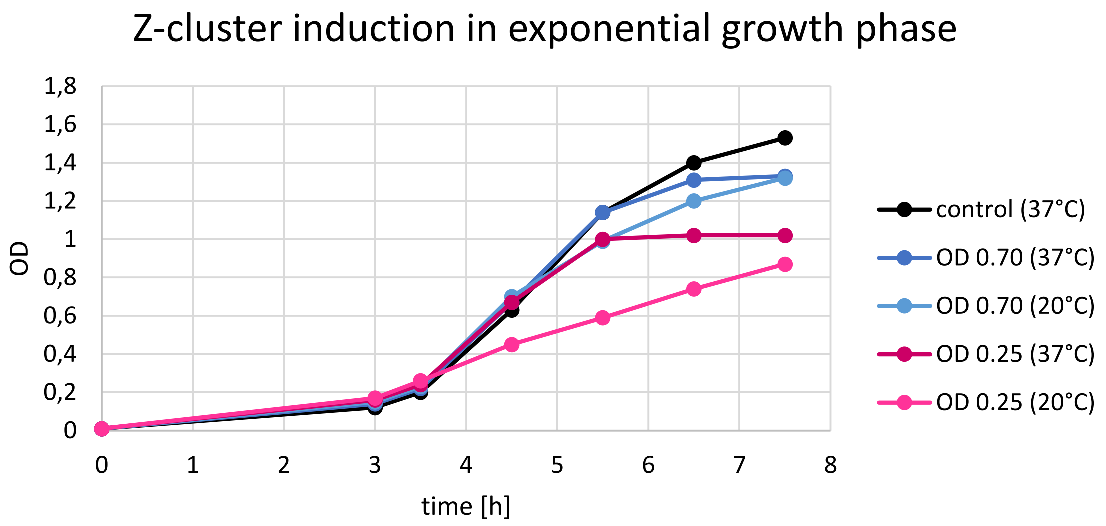

Supplementary Figure 11. Toxic effect of the expressed Z-cluster on *E. coli* growth. Bacterial growth was followed by measuring the optical density (OD) of liquid colonies. Cultures were induced with low concentration of IPTG (84 µM) at two points of the exponential growth phase (OD 0.25 or 0.70) and incubated at 20 or 37°C afterwards (legend to the right). All cultures showed arrested growth in a matter of a few generations compared to a non-induced control.

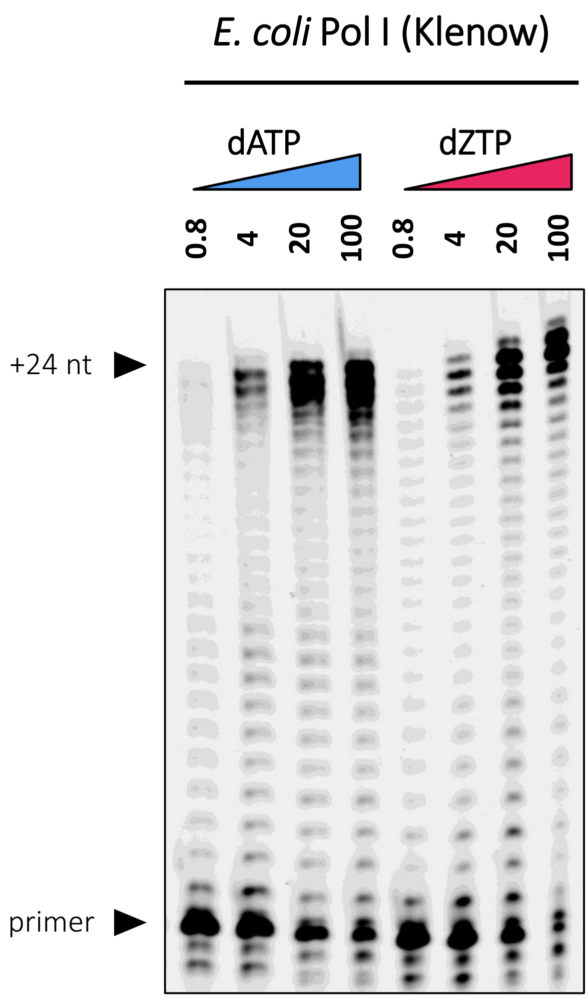

Supplementary Figure 12. Results of DNA polymerase activity tests of *E. coli* Pol I (Klenow fragment). dATP (blue) or dZTP (magenta) are used as the substrate dNTP on a polythymine (dT_{24}) template. Nucleotide concentrations are given in µM under the triangles. Full-length complementary strand appears for the same concentrations of dATP and dZTP.

**Protein structure**	**PurZ + dGMP, dATP**	**MazZ + dGDP, Mn^{2+}**
PDB ID	7ODX	7ODY
Cell parameters		
Space group	P 6_2 2 2	P 2_1 2_1 2_1
a, *b*, *c* (Å)	108.18, 108.18, 142.33	53.59, 91.37, 114.28
Solvent content (%)	58.8	56.1
Data statistics		
Resolution (Å)	44.50 - 1.70<br>(1.74 - 1.70)	48.52 - 1.43<br>(1.47 - 1.43)
Wavelength (Å)	0.9801	1.1271
Rmerge (%)	8.1 (266.1)	8.9 (193.8)
Completeness (%)	99.8 (98.0)	99.9 (98.3)
Multiplicity	39.6 (39.4)	13.1 (10.9)
$I/\sigma(I)$	34.1 (1.7)	14.1 (1.1)
$CC_{1/2}$	1.000 (0.694)	0.999 (0.579)
Refinement		
Resolution (Å)	42.33 - 1.70	48.45 - 1.43
Unique reflections	54,792	104,219
R_{work}/R_{free} (%)	15.94/17.60	16.44/16.65
No. of non-hydrogen atoms		
Protein	2702	2937
Ligand	53	168
Ions	0	52
Water	394	488
Hydrogen atoms	No	Yes
Protein geometry		
RMSD - bond lengths (Å)	0.010	0.008
RMSD – bond angles (°)	1.00	0.89

**Protein structure**	**PurZ + dGMP, dATP**	**MazZ + dGDP, Mn^{2+}**
PDB ID	7ODX	7ODY
Protein geometry (cont.)		
Ramachandran favored/outliers (%)	96.84/0.00	98.92/0.00
Rotamers favored/poor (%)	98.24/0.00	97.00/0.00
Clashscore	2.57	3.23
B-factors ($Å^2$)		
Type	Anisotropic	Anisotropic
TLS	1 group	1 group
Protein	30.71	24.76
Ligand	28.27	18.93
Ions	-	37.75
Water	43.73	38.67

Supplementary Table 1. Diffraction data collection and Model Refinement statistics. Numbers in parenthesis refer to the highest-resolution shell.

Protein name	Function	Start	End
DatZ	dATP triphosphohydrolase	13934	13407
MazZ	(d)GTP diphosphohydrolase	14248	13931
PurZ	N6-succino 2-amino deoxyadenylate synthetase	15308	14229
Exonuclease VII	exonuclease	17970	17011
MarR	transcription repressor*	18735	18382
Helicase SF2	helicase*	18872	20137
VRR nuclease	resolvase*	20147	20656
PrimPol	DNA polymerase	20825	23038

Supplementary Table 2. Position of replication-related protein genes on the new, S-2L genome sequence with high-coverage (MW334946). Genes *datZ*, *mazZ* and *purZ* are encoded in a highly compact way, overlapping at their very ends. Proteins with functions marked with an asterisk (*) have not been tested experimentally; their role is predicted by homology.

Gene	Nucleotide sequence
datZ	ATGACACTCCAGATCACCGAGACCTACGAGCGCCTGAGGGCGTCCCACATCAGCCGGTGGGGATCGTCCAGACGACCTACCCGCAGAA<br>CATCGCCGAACACATGTGGCGCGTTTGGCTCCTGTGCCGGGACTGGGGCGCTGCCGCCGGCATGCCCCAGCACACGGTCCGCCAGGCCT<br>GCGAGTTTGCCCTGGTCCACGACCTGGCCGAGATCCGGACGGGCGACGCCCCGACGCCCCACAAGACCCCGGAGCTCAAGGAGCTCCTG<br>GCCGGCATCGAGGCCCAGATCGTCCCGGAGGTGGCCGAGCTCGAGGCGACCATGGCCCCGGAGGCCAGAGAGCTTTGGAAGTTCTGCGA<br>CACCGCCGAGGCCGTCCTGTTCCTCAAGGTCAACGGCCTGGGCGCCCACGCCTACGACGTCCAGCACCTGCTGATGGAGCAGATGAAAC<br>GGCGCCTGATGGACTCGGTGTTGGATGTGGAGGTGCAGGACGAGCTCATGTTCCAGTTCGAGCGGACGATCAAGAAGACGTGA
mazZ	ATGCCCGCTACCGTCGCTGAGCTCCAGGCGGAGATCGCCGCCTGGATCCACCCCCTGAACCCCGACCGCCGCCCCGGCGGCACCATCGC<br>CAAGCTCCTGGAGGAGATCGGGGAGTTGATCGCCAGCGACCGGGCCCACGACCCGCTCGAGGTGGCCGACGTCCTGATCCTGGCCCTCG<br>ACCTGGCGACGCTCCTGGGCGTCGACGTGACCGAGGCCATCCGCGCCAAGCTCGCCATCAACCGGGCCCGCTCCTGGGCCCGAGCCGAT<br>AACGGCGCCATGCGCCACATCCCCGGTTCCGATACCCCCTCCTTCCCATGA
purZ	ATGCTGTCCATTCCCCCCTACTATCGCGTGAAGAACTGCAACCTGATCGTCGACTGCCAGTACGGCAGCACCGGCAAGGGGCTCCTGGC<br>CGGCTACCTGGGGGCGCTCGAGGCCCCGCAGGTGCTGTGCATGGCACCCAGCCCCAACGCCGGCCACACCCTGGTCGAGGAGGACGGCA<br>CCGCCCGCGTCCACAAGATGCTGCCCCTGGGCATCACCAGCCCCAGCCTTGAGCGGATCTACCTGGGCCCCGGCTCGGTGATCGACATG<br>GACCGGCTCCTAGAGGAGTACCTGGCCCTCCCCCGGCAGGTGGAGCTCTGGGTCCACCAGAACGCCGCCGTCGTCCTCCAGGAGCACCG<br>GGATGAGGAGGCCGCCGGGGGCCTGGCCCCAGGCTCGACCCGCAGCGGCGCCGGCTCGGCGTTTATCGCCAAGATCCGCCGCCGCCCTG<br>GGACGCTCCTGTTCGGTGAGGCCGTCCGGGATCACCCGCTCCACGGTGTCGTCCGGGTCGTCGACACCCGGACCGCCCAGGACATGCTG<br>TTTCGGACCCGGTCGATCCAGGCCGAGGGGTGCCAGGGCTACAGCCTGTCGGTCCACCACGGGGCCTACCCCTACTGCACCGCCCGGGA<br>CGTCACGACGGCCCAGCTGATCGCCGACTGCGGCCTGCCCTACGACGTCGCCCGGATCGCCCGGGTCGTCGGCTCGATGCGGACCTACC<br>CGATCCGGGTGGCCAACCGCCCGGAGGCCGGTGAGTGGAGCGGCCCCTGCTACCCCGACTCGGTCGAGTGCCAGTTCGCCGACCTGGGC<br>CTGGAGCAGGAGTACACCACCGTGACGAAGCTCCCCCGCCGGATCTTTACGTTTAGCGCCATCCAGGCGCACGAGGCCATCGCCCAGAA<br>CGGCGTGGACGAGGTGTTCCTCAACTTCGCCCAGTACCCGCCCAGCCTCGGGGCTCTCGAGGACATCCTCGACGCCATCGAGGCCAGGG<br>CGGAGGTGACCTACGTCGGCTTCGGCCCGAAGGTCACCGACGTCTACCACACCCCCACCCGGGCAGAGCTCGAAGGTTTGTATGCCCGC<br>TACCGTCGCTGA
pplA	ATGTCAACCCCCGCACCAGCCTTCGACCGGACCAGATCCTCCTCCACCTGTCGCTCCTCCGGAAGGACATCGCCACGACCCGGTACCG<br>GGCGATCTGGCCCAGGCGAGAGGACAAGGTAAAAGCCTGGACGACGCCCCTGACCGGGGCCACGGTCCAGGACGCCGTCACCCAGGGAT<br>TCAACAGCTACATCGTCGTAGGCGACGGCGGCGACTCCGACGCCGAGATCACCAGTGTCAACGCCATCTTCGGCGAGTGGGACGACGGC<br>GACCTGGCCTGGCAGGTCGGCGCCTGGGAGGCCTGCGGCCTGCCGCGGCCGAGCTTCCAGCTGCGCACCGGGGGCAAGTCGATCCACCA<br>CTACTGGGTGTTCCACAGCCCTGTGGACGTCCCGGCCTGGACCGAGCTCCAGGCCCGCCTGATCGCCCTGGCCGGCTTCGACACGACGA<br>ACCGGAACCCCTCCCGGGTGATGCGCCTGGCCGGCTGCCCCCACCAGCGCACCGGGGAGGTGGCCCAGATCTTCAACGCGACCGGGGAG<br>CTCTACGACCCCGGGCAGATGCTGCAGGTCCTGCCCCCGGTGCCGATCGACCCGCCGGCTGCCGCCCCGGTGGCCCCGGGAGGTGCCCC<br>CAGTTCGATGGACGACATCCGGGCCGCCCTGGCCCAGATCCCACCCCGTCCCGGGGCAGGGAGCGGCACCTACGCCGAGTACCGCAACA<br>TCCTCTGGGGCCTGGTTAAGGCCGTCGAGGAGGCCGGCGGCACCCGGGACCAGGCCGTGGCCATGATGCAGGCGCACAGCCCCGAGGGC<br>TGGGATTGCGCCCAGGTGGCCCGCTCCGGGGGCAAGAAGATCAGCACCGGGACGTTCTGGTGGCATGCGATGTCCTACGGCTGGGCACC<br>GCCGAAGAAGGCCCCGGAGCCGCCGCCCCAGGCCCGCCAGGTGCCGGCCGTGGCCGCCGTGCTCCAGGCCGCAGAGGCCGCCCCTGGAA<br>CCGGCACCGAGCACGGCCCCTGGGCGCCGCTGCCCCCGGGCTGGCAGGGCACGAACAAGGAGGGCCTGCCCAGGGCCTCGCAGATCACC<br>ACCTACGAACTGGCCCTGCTGATGCAGGTCTCCCTGCGGGGGGTGCTCTGGCACAACGAGATGTCAGGCGAAGTCATGCACGGCAAGAC<br>GGCCCTCTCGCCGATCGAGCTCCAGATCGCCTACAGCCGGCTCGAGGGCCTCGGCTACAAGGTCACCAAGGAGAACGCCAAGACCGCCA<br>TCCTGCAGGCGTCGATCGCCGACCTGCGGCACCCCGTCCGGGAGTACCTCAACACCTGCACGACGCCCCTGCCCGACGAGGTCTGGGCC<br>GACATCGCCAACGCCCTGCTGGGCCCCGGGCACAGCGCGTTCGACTCCAGCGCCATCCGCAAGTGGCTGATCTTCGCCGTGGCCCGGGT<br>CTTCCAGCCCGGCTGCCCCTTCGGCTTCATGCTGGTGCTGGCTGGCGCCCAGCAGATGCACAAGACCCGGTTCTTTAACACCCTGGCCT<br>CAGACGAGTGGTTCCTGGGCGGATTCCAGCGGGGCCGCTCTGACACCGACGACCTGATTGCCCTGCACCGGTCCTGGATCACCGAGTGG<br>GGGGAGCTCGACGGCGGCCTCTCCAAGCACGACAGCGCCGAGCTCAAGGCGATGATCGACCGGAAGGTGGACGTGCTCCGGAGGCCCTA<br>CGCCGCCACGCACGAAAGCTGCCCCCGGAGCTTCGTCCTCTGCGGGACGACGAACCGCCGGGATGGGCTCTTCACCGACCCGACCGGCA<br>ACAGGCGGTACGTGGTCGTGCCCGTCAACCAGCGGATCGACAGCGAGCGCCTGGAGCAGATGCGAGACCAGATCTGGGCAACCGCCCTC<br>CGGGAGTACCGCAGCGGCAAGCTCTGGTACCTCGACGAGGAGGAGCTGGAGATCAACGCGAAACGCAACAAGGGCCTTGAGGTGGAGGA<br>CGCCTGGGTGGGGACGATCCAGATGCACCTGAATAGCTCGATCGACCTGGAGCGGCTGACCGACGGGCGCTACGGCATCAACATCGAGT<br>CAGTCTACCTCAAGATCGAGCCCGAGGTGGACGCCGTGGCCCGGGCTTCGGAAAGCGGATCCGGGACACCATGCTGAGCCTGGGCTGG<br>GAGCCCGTGCGGCTGCGTCTCGCCAGCGACCCGAGCGGCAACCCGGTGAGGCGTTGGGCGCCCGTCCAGGGGGGGTAG

Supplementary Table 3. Nucleotide sequences of *datZ*, *mazZ*, *purZ* and *pplA* native genes (GenBank MW334946).

Gene	Nucleotide sequence
datZ	ATGACACTGCAGATTACCGAAACCTATGAACGTCTGCGTGCAAGCCATATTAGCCGTTGGGGTATTGTTCAGACCACCTATCCGCAGAA<br>TATTGCAGAACATATGTGGCGTGTTTGGCTGCTGTGTCGTGATTGGGGTGCAGCAGCAGGTATGCCGCAGCATACAGTTCGTCAGGCAT<br>GTGAATTTGCACTGGTTCATGATCTGGCAGAAATTCGTACCGGTGATGCACCGACACCGCATAAAACACCGGAACTGAAAGAACTGCTG<br>GCAGGTATTGAAGCACAGATTGTTCCGGAAGTTGCAGAACTGGAAGCAACCATGGCACCGGAAGCACGTGAACTGTGGAAATTTTGTGA<br>TACCGCAGAAGCAGTTCTGTTCCTGAAAGTTAATGGTCTGGGTGCACATGCATATGATGTTCAGCATCTGCTGATGGAACAAATGAAAC<br>GTCGTCTGATGGATAGCGTTCTGGATGTTGAAGTTCAGGATGAACTGATGTTTCAGTTTGAACGCACCATCAAAAAGACCTAA
mazZ	ATGCCTGCAACCGTTGCCGAACTGCAGGCAGAAATTGCAGCCTGGATTCATCCGCTGAATCCGGATCGTCGTCCTGGTGGCACCATTGC<br>AAAACTGCTGGAAGAAATCGGTGAACTGATTGCAAGCGATCGTGCACATGATCCGCTGGAAGTTGCAGATGTTCTGATTCTGGCACTGG<br>ATCTGGCAACCCTGCTGGGTGTTGATGTTACCGAAGCAATTCGTGCCAAACTGGCAATTAATCGTGCACGTAGCTGGGCACGTGCAGAT<br>AATGGTGCAATGCGTCATATTCCGGGTAGCGATACCCCGAGCTTTCCGTAA
purZ	ATGCTGAGCATTCCGCCTTATTATCGTGTGAAAAATTGCAACCTGATTGTGGATTGTCAGTATGGTAGCACCGGTAAAGGTCTGCTGGC<br>AGGTTATCTGGGTGCACTGGAAGCACCGCAGGTTCTGTGTATGGCACCGAGTCCGAATGCAGGTCATACCCTGGTTGAAGAGGATGGCA<br>CCGCACGTGTTCATAAAATGCTGCCGCTGGGTATTACCAGTCCGAGCCTGGAACGTATTTATCTTGGTCCGGGTAGCGTTATTGATATG<br>GATCGTCTGCTGGAAGAATATCTGGCACTGCCTCGTCAGGTTGAACTGTGGGTTCATCAGAATGCAGCAGTTGTTCTGCAAGAACATCG<br>TGATGAAGAAGCAGCAGGCGGTCTGGCACCGGGTAGCACCCGTAGCGGTGCAGGTAGCGCATTTATTGCAAAAATTCGTCGTCGTCCGG<br>GTACACTGCTGTTTGGTGAAGCAGTTCGTGATCATCCGCTGCATGGTGTTGTTCGTGTTGTTGATACCCGTACCGCACAGGATATGCTG<br>TTTCGTACCCGTAGCATTCAGGCAGAAGGTTGTCAGGGTTATAGCCTGAGCGTTCATCATGGTGCATATCCGTATTGTACAGCACGTGA<br>TGTTACCACCGCACAGCTGATTGCAGATTGTGGTCTGCCGTATGATGTTGCACGTATTGCACGTGTTGTGGGTAGCATGCGTACCTATC<br>CGATTCGTGTTGCAAATCGTCCGGAAGCCGGTGAATGGTCAGGTCCGTGTTATCCGGATTCAGTTGAATGTCAGTTTGCAGATCTGGGC<br>CTTGAACAAGAATATACCACCGTTACCAAACTGCCACGTCGCATTTTTACCTTTAGCGCAATTCAGGCACATGAAGCAATTGCACAGAA<br>TGGTGTTGATGAAGTGTTTCTGAATTTTGCACAGTATCCGCCTAGCCTGGGAGCCCTGGAAGATATTCTGGATGCAATTGAAGCACGTG<br>CCGAAGTTACCTATGTTGGTTTTGGTCCGAAAGTTACCGATGTGTATCATACCCCGACACGTGCAGAACTGGAAGGTCTGTATGCACGT<br>TATCGTCGTTAA
pplA	ATGAGCACACCGGCACCGGCATTTGATCGTGATCAGATTCTGCTGCATCTGAGCCTGCTGCGTAAAGATATTGCAACCACACGTTATCG<br>TGCAATTTGGCCTCGTCGTGAAGATAAAGTTAAAGCATGGACCACACCGCTGACCGGTGCAACCGTTCAGGATGCAGTTACCCAGGGTT<br>TTAATAGCTATATCGTTGTTGGTGATGGTGGTGATAGTGATGCAGAAATTACCAGCGTTAATGCCATTTTTGGTGAATGGGATGATGGT<br>GATCTGGCATGGCAGGTTGGTGCATGGGAAGCATGTGGTCTGCCTCGTCCGAGCTTTCAGCTGCGTACCGGTGGTAAAAGCATTCATCA<br>TTATTGGGTTTTTCACAGTCCGGTTGATGTTCCGGCATGGACCGAACTGCAGGCACGTCTGATTGCACTGGCAGGTTTTGATACCACCA<br>ATCGTAATCCGAGCCGTGTTATGCGTCTGGCAGGCTGTCCGCATCAGCGCACCGGTGAAGTTGCACAGATTTTCAATGCAACCGGTGAA<br>CTGTATGATCCGGGTCAGATGCTGCAGGTTCTGCCTCCGGTTCCGATTGATCCGCCTGCAGCAGCTCCGGTTGCGCCTGGTGGTGCACC<br>GAGCAGCATGGATGATATTCGTGCAGCACTGGCACAGATTCCGCCTCGTCCTGGTGCAGGTAGCGGCACCTATGCAGAATATCGTAATA<br>TTCTGTGGGGTTTAGTTAAAGCCGTTGAAGAGGCAGGCGGTACACGTGATCAGGCAGTTGCAATGATGCAGGCACATAGTCCGGAAGGT<br>TGGGATTGTGCACAGGTTGCACGTAGTGGTGGCAAAAAAATCAGCACCGGTACATTTTGGTGGCATGCAATGAGCTATGGTTGGGCACC<br>GCCTAAAAAAGCACCGGAACCGCCTCCGCAGGCACGCCAGGTTCCAGCAGTTGCAGCAGTTCTGCAGGCAGCAGAAGCAGCCCCTGGTA<br>CAGGCACCGAACATGGTCCGTGGGCTCCGCTGCCTCCTGGTTGGCAGGGCACCAATAAAGAAGGTCTGCCACGCGCAAGCCAGATTACC<br>ACCTATGAACTGGCACTGCTGATGCAGGTTAGCCTGCGTGGTGTTCTGTGGCATAATGAAATGAGCGGTGAAGTAATGCATGGTAAAAC<br>CGCACTGAGCCCGATTGAACTGCAGATTGCATATAGCCGTCTGGAAGGTCTGGGTTATAAAGTGACCAAAGAAAATGCAAAAACCGCAA<br>TCCTGCAGGCAAGCATTGCCGATCTGCGTCATCCGGTTCGTGAATATCTGAATACCTGTACAACCCCTCTGCCGGATGAAGTTTGGGCA<br>GATATTGCCAATGCACTGTTAGGTCCGGGTCATAGCGCATTTGATAGCAGCGCAATTCGTAAATGGCTGATCTTTGCAGTTGCACGTGT<br>TTTTCAGCCTGGTTGTCCGTTTGGTTTTATGCTGGTGCTGGCAGGCGCACAGCAGATGCATAAAACACGCTTTTTTAACACCCTGGCAT<br>CCGATGAATGGTTTTTAGGTGGTTTTCAGCGTGGTCGTAGCGATACCGATGATCTGATTGCCCTGCATCGTAGCTGGATTACCGAATGG<br>GGTGAACTGGATGGTGGTCTGAGCAAACATGATAGCGCAGAACTGAAAGCAATGATTGATCGTAAAGTTGATGTGCTGCGTCGTCCGTA<br>TGCAGCAACCCATGAAAGCTGTCCGCGTAGCTTTGTTCTGTGTGGTACAACCAATCGTCGTGATGGTCTGTTTACCGATCCGACCGGTA<br>ATCGTCGTTATGTTGTTGTTCCGGTTAATCAGCGTATTGATAGCGAACGTCTGGAACAAATGCGCGATCAGATTTGGGCCACCGCACTG<br>CGCGAATATCGTTCAGGTAAACTGTGGTATCTGGATGAAGAGGAACTGGAAATTAATGCCAAACGCAATAAAGGTCTGGAAGTTGAAGA<br>TGCATGGGTTGGCACCATTCAGATGCACCTGAACAGCAGCATTGATCTGGAACGTCTGACCGATGGTCGTTATGGTATTAACATTGAAA<br>GCGTGTACCTGAAAATTGAACCGGAAGTTGGTCGTCGTGGTCCTGGTTTTGGTAAACGTATTCGTGATACCATGCTGAGCTTAGGTTGG<br>GAACCTGTTCGTCTGCGCCTGGCAAGCGATCCGAGTGGTAATCCGGTGCGTCGCTGGGCACCTGTTCAAGGTGGTTAA

Supplementary Table 4. Nucleotide sequences of *datZ*, *mazZ*, *purZ* and *pplA* codon-optimized genes.

Protein	Protein sequence
DatZ	MTLQITETYERLRASHISRWGIVQTTYPQNIAEHMWRVWLLCRDWGAAAGMPQHTVRQACEFALVHDLAEIRTGDAPTPHKTPELK<br>ELLAGIEAQIVPEVAELEATMAPEARELWKFCDTAEAVLFLKVNGLGAHAYDVQHLLMEQMKRRLMDSVLDVEVQDELMFQFERTI<br>KKT-
MazZ	MPATVAELQAEIAAWIHPLNPDRRPGGTIAKLLEEIGELIASDRAHDPLEVADVLILALDLATLLGVDVTEAIRAKLAINRARSWA<br>RADNGAMRHIPGSDTPSFP-
PurZ	MLSIPPYYRVKNCNLIVDCQYGSTGKGLLAGYLGALEAPQVLCMAPSPNAGHTLVEEDGTARVHKMLPLGITSPSLERIYLGPGSV<br>IDMDRLLEEYLALPRQVELWVHQNAAVVLQEHRDEEAAGGLAPGSTRSGAGSAFIAKIRRRPGTLLFGEAVRDHPLHGVVRVVDTR<br>TAQDMLFRTRSIQAEGCQGYSLSVHHGAYPYCTARDVTTAQLIADCGLPYDVARIARVVGSMRTYPIRVANRPEAGEWSGPCYPDS<br>VECQFADLGLEQEYTTVTKLPRRIFTFSAIQAHEAIAQNGVDEVFLNFAQYPPSLGALEDILDAIEARAEVTYVGFGPKVTDVYHT<br>PTRAELEGLYARYRR-
PrimPol	MSTPAPAFDRDQILLHLSLLRKDIATTRYRAIWPRREDKVKAWTTPLTGATVQDAVTQGFNSYIVVGDGGDSDAEITSVNAIFGEW<br>DDGDLAWQVGAWEACGLPRPSFQLRTGGKSIHHYWVFHSPVDVPAWTELQARLIALAGFDTTNRNPSRVMRLAGCPHQRTGEVAQI<br>FNATGELYDPGQMLQVLPPVPIDPPAAAPVAPGGAPSSMDDIRAALAQIPPRPGAGSGTYAEYRNILWGLVKAVEEAGGTRDQAVA<br>MMQAHSPEGWDCAQVARSGGKKISTGTFWWHAMSYGWAPPKKAPEPPPQARQVPAVAAVLQAAEAAPGTGTEHGPWAPLPPGWQGT<br>NKEGLPRASQITTYELALLMQVSLRGVLWHNEMSGEVMHGKTALSPIELQIAYSRLEGLGYKVTKENAKTAILQASIADLRHPVRE<br>YLNTCTTPLPDEVWADIANALLGPGHSAFDSSAIRKWLIFAVARVFQPGCPFGFMLVLAGAQQMHKTRFFNTLASDEWFLGGFQRG<br>RSDTDDLIALHRSWITEWGELDGGLSKHDSAELKAMIDRKVDVLRRPYAATHESCPRSFVLCGTTNRRDGLFTDPTGNRRYVVVPV<br>NQRIDSERLEQMRDQIWATALREYRSGKLWYLDEEELEINAKRNKGLEVEDAWVGTIQMHLNSSIDLERLTDGRYGINIESVYLKI<br>EPEVGRRGPGFGKRIRDTMLSLGWEPVRLRLASDPSGNPVRRWAPVQGG-

Supplementary Table 5. Protein sequences of DatZ, MazZ, PurZ and PrimPol.

Oligonucleotide name	Sequence (5'-3')
T_{24} overhang template	TTTTTTTTTTTTTTTTTTTTTTTTAACAAGGCTAATGCG
Primer	CGCATTAGCCTTGTT

Supplementary Table 6. Oligonucleotides used for the polymerase assay.
