# Supplementary material for: Characterization of a triad of genes in cyanophage S-2L sufficient to replace adenine by 2-aminoadenine in bacterial DNA
Source: Nat Commun. 2021 Aug 5;12:4710. doi: 10.1038/s41467-021-25064-x (PMC8342488; doi:10.1038/s41467-021-25064-x)
Supplement: Supplementary file 2 — Reporting Summary [file 41467_2021_25064_MOESM2_ESM.pdf]

nature research

Corresponding author(s): Marc Delarue

Last updated by author(s): Jul 12, 2021

Reporting Summary

Nature Research wishes to improve the reproducibility of the work that we publish. This form provides structure for consistency and transparency in reporting. For further information on Nature Research policies, see our Editorial Policies and the Editorial Policy Checklist.

Statistics

For all statistical analyses, confirm that the following items are present in the figure legend, table legend, main text, or Methods section.

n/a	Confirmed	
☐	☒	The exact sample size (*n*) for each experimental group/condition, given as a discrete number and unit of measurement
☐	☒	A statement on whether measurements were taken from distinct samples or whether the same sample was measured repeatedly
☒	☐	The statistical test(s) used AND whether they are one- or two-sided<br>*Only common tests should be described solely by name; describe more complex techniques in the Methods section.*
☒	☐	A description of all covariates tested
☒	☐	A description of any assumptions or corrections, such as tests of normality and adjustment for multiple comparisons
☒	☐	A full description of the statistical parameters including central tendency (e.g. means) or other basic estimates (e.g. regression coefficient) AND variation (e.g. standard deviation) or associated estimates of uncertainty (e.g. confidence intervals)
☒	☐	For null hypothesis testing, the test statistic (e.g. *F*, *t*, *r*) with confidence intervals, effect sizes, degrees of freedom and *P* value noted<br>*Give P values as exact values whenever suitable.*
☒	☐	For Bayesian analysis, information on the choice of priors and Markov chain Monte Carlo settings
☒	☐	For hierarchical and complex designs, identification of the appropriate level for tests and full reporting of outcomes
☒	☐	Estimates of effect sizes (e.g. Cohen's *d*, Pearson's *r*), indicating how they were calculated

Our web collection on statistics for biologists contains articles on many of the points above.

Software and code

Policy information about availability of computer code

Data collection	Software for crystallographic data collection and reduction: xds (2020 annual release), ccp4 7.1. This software is public software, freely distributed. It is properly referenced in the list of References. We have not used any custom algorithm or software.
Data analysis	Software for model building and refinement of crystallographic structures: ccp4 7.1, Coot 0.8.9 and phenix 1.19.2. This software is public software, freely distributed. It is properly referenced in the list of References. We have not used any custom algorithm or software.<br>For sequence analysis we have used minimap2 v2.17, freebayes v1.3.2, vcflib v0.1.0 and of course Blast, as available from the NCBI web server.

For manuscripts utilizing custom algorithms or software that are central to the research but not yet described in published literature, software must be made available to editors and reviewers. We strongly encourage code deposition in a community repository (e.g. GitHub). See the Nature Research guidelines for submitting code & software for further information.

Data

Policy information about availability of data

All manuscripts must include a data availability statement. This statement should provide the following information, where applicable:

- Accession codes, unique identifiers, or web links for publicly available datasets
- A list of figures that have associated raw data
- A description of any restrictions on data availability

The NGS sequence of cyanophage S-2L is deposited in the GenBank database, under the accession code MW334946 [https://www.ncbi.nlm.nih.gov/nuccore/

MW334946.1]. The crystallographic data for cyanophage S-2L proteins PurZ and MazZ bound to their respective ligands are deposited in the Protein Data Bank (PDB) under the accession codes 7ODX [https://doi.org/10.2210/pdb7ODX/pdb] and 7ODY [https://doi.org/10.2210/pdb7ODY/pdb]. Source data are provided with this paper in the Source Data file. Other data are available from the corresponding author upon request.

Field-specific reporting

Please select the one below that is the best fit for your research. If you are not sure, read the appropriate sections before making your selection.

- [x] Life sciences
- [ ] Behavioural & social sciences
- [ ] Ecological, evolutionary & environmental sciences

For a reference copy of the document with all sections, see nature.com/documents/nr-reporting-summary-flat.pdf

Life sciences study design

All studies must disclose on these points even when the disclosure is negative.

Sample size	For diffraction data, 5-6 crystals were screened on a synchrotron beam line; they had similar diffraction properties and only the best complete dataset was kept.
Data exclusions	No data was excluded
Replication	All biochemical tests were done at least twice. All attempts to replicate data were successful. See above for crystallographic data.
Randomization	Not applicable
Blinding	Not applicable

Reporting for specific materials, systems and methods

We require information from authors about some types of materials, experimental systems and methods used in many studies. Here, indicate whether each material, system or method listed is relevant to your study. If you are not sure if a list item applies to your research, read the appropriate section before selecting a response.

Materials & experimental systems

n/a	Involved in the study	
[x]	[ ]	Antibodies
[x]	[ ]	Eukaryotic cell lines
[x]	[ ]	Palaeontology and archaeology
[x]	[ ]	Animals and other organisms
[x]	[ ]	Human research participants
[x]	[ ]	Clinical data
[x]	[ ]	Dual use research of concern

Methods

n/a	Involved in the study	
[x]	[ ]	ChIP-seq
[x]	[ ]	Flow cytometry
[x]	[ ]	MRI-based neuroimaging
